# Milk oligosaccharide-driven persistence of *Bifidobacterium pseudocatenulatum* modulates local and systemic microbial metabolites upon synbiotic treatment in conventionally colonized mice

**DOI:** 10.1186/s40168-023-01624-9

**Published:** 2023-08-28

**Authors:** Jules A. Larke, Britta E. Heiss, Amy M. Ehrlich, Diana H. Taft, Helen E. Raybould, David A. Mills, Carolyn M. Slupsky

**Affiliations:** 1 Department of Nutrition, University of California, Davis, One Shields Avenue, Davis, CA 95616 USA; 2 Department of Food Science and Technology, University of California, Davis, One Shields Avenue, Davis, CA 95616 USA; 3 Department of Anatomy, Physiology, and Cell Biology, School of Veterinary Medicine, University of California, Davis, , Davis, CA USA

**Keywords:** Probiotic, Prebiotic, Colonization, HMO, Human milk oligosaccharide, 2′-FL, Mice

## Abstract

**Background:**

Bifidobacteria represent an important gut commensal in humans, particularly during initial microbiome assembly in the first year of life. Enrichment of *Bifidobacterium* is mediated though the utilization of human milk oligosaccharides (HMOs), as several human-adapted species have dedicated genomic loci for transport and metabolism of these glycans. This results in the release of fermentation products into the gut lumen which may offer physiological benefits to the host. Synbiotic pairing of probiotic species with a cognate prebiotic delivers a competitive advantage, as the prebiotic provides a nutrient niche.

**Methods:**

To determine the fitness advantage and metabolic characteristics of an HMO-catabolizing *Bifidobacterium* strain in the presence or absence of 2′-fucosyllactose (2′-FL), conventionally colonized mice were gavaged with either *Bifidobacterium pseudocatenulatum* MP80 (*B.p.* MP80) (as the probiotic) or saline during the first 3 days of the experiment and received water or water containing 2′-FL (as the prebiotic) throughout the study.

**Results:**

16S rRNA gene sequencing revealed that mice provided only *B.p.* MP80 were observed to have a similar microbiota composition as control mice throughout the experiment with a consistently low proportion of *Bifidobacteriaceae* present. Using ^1^H NMR spectroscopy, similar metabolic profiles of gut luminal contents and serum were observed between the control and *B.p.* MP80 group. Conversely, synbiotic supplemented mice exhibited dramatic shifts in their community structure across time with an overall increased, yet variable, proportion of *Bifidobacteriaceae* following oral inoculation. Parsing the synbiotic group into high and moderate bifidobacterial persistence based on the median proportion of *Bifidobacteriaceae*, significant differences in gut microbial diversity and metabolite profiles were observed. Notably, metabolites associated with the fermentation of 2′-FL by bifidobacteria were significantly greater in mice with a high proportion of *Bifidobacteriaceae* in the gut suggesting metabolite production scales with population density. Moreover, 1,2-propanediol, a fucose fermentation product, was only observed in the liver and brain of mice harboring high proportions of *Bifidobacteriaceae*.

**Conclusions:**

This study reinforces that the colonization of the gut with a commensal microorganism does not guarantee a specific functional output.

Video Abstract

**Supplementary Information:**

The online version contains supplementary material available at 10.1186/s40168-023-01624-9.

## Introduction

Early, dominant, infant borne *Bifidobacterium* colonization in breastfed infants is favored by the consumption of human milk oligosaccharides (HMOs) due to their prebiotic nature [1]. These structurally-complex oligosaccharides comprised of a range of monomers and linkages [2, 3] establish a nutrient niche in the gut that selectively enrich several *Bifidobacterium* species [4, 5]. Isolated species associated with breastfeeding and capable of HMO metabolism include *B. longum* subsp. *infantis*, *B. longum* subsp. *longum*, *B. breve*, and *B. pseudocatenulatum* [4, 5]*.* In infant cohort studies, associations between HMO degradation, enrichment of select *Bifidobacterium* species, higher fecal acetate and lactate, and advantageous health outcomes have been observed [6–11]. As such, robust colonization of *Bifidobacterium* during infancy has been linked with improved markers for type 1 diabetes [12], reduced likelihood of obesity [13, 14], robust vaccine responses [15], and lower antimicrobial resistance gene carriage [8, 16, 17].

Given the associations of *Bifidobacterium longum* and health outcomes in infants, there is an increased interest to promote *Bifidobacterium* populations in later human life stages and model colonization of this organism to scrutinize mechanisms of action. Probiotic supplementation is a commonly used strategy to manipulate the gut microbiota; however, efficacy is influenced by inter-individual variation of host related factors including genetics, diet, and microbiome composition [18–22]. As such, deriving health benefits from the probiotic may be case specific in which only certain diet and/or microbiome configurations promote metabolic or other microbial activities responsible for the benefit [23]. While probiotic bacteria are often capable of surviving passage through the gastrointestinal tract, most probiotics do not colonize, and little is known about their interaction with indigenous microbiota and gut accessible nutrient resources. However, synergistic synbiotics comprised of prebiotics selectively utilized by a co-administered probiotic may act to enhance colonization and functionality in the gut [24]. With this targeted enrichment strategy, there is a greater likelihood of colonization through which health outcomes can be achieved.

The persistence of a species is defined as the time between its emergence and extinction within a region [25]. Nutrient availability, environmental conditions, and competition between species influences whether a microbe persists. Bacterial persistence consists of a microbe replicating at an equal or greater rate than washout [26]. A prior synbiotic rodent model pairing a fermented milk product and five food-borne bacterial strains found that the supplemented bacteria were quantifiable in the feces during the feeding periods with only a subset of rats continuing to shed one, *Lactococcus lactis* subsp. *lactis*, of the five strains 2 days post-supplementation [27]. Researchers concluded that a subset of rats were permissive to probiotic persistence while others were resistant. Alternatively, by exploiting the established, evolutionary-selected, complementary milk glycan-bacterial synbiotic pairing, we established a persistent population of *Bifidobacterium pseudocatenulatum* MP80 (*B.p*. MP80), a breastfed infant bacterial isolate, with continuous supplementation of the HMO 2′-fucosyllactose (2′-FL) [28]. Strain selection was based on *B.p.* MP80’s capability to rapidly grow on and metabolize 2′-FL [29]. When mice were subjected to a chemically induced colitis model, synbiotic treatment improved health outcomes and reduced inflammation suggesting a synergistic protective effect. Given the evidence of *Bifidobacterium* engraftment among probiotic supplemented breastfed infants [30, 31], this mouse persistence model was designed to recapitulate the critical role HMOs play in the colonization of HMO-consuming *Bifidobacterium*.

Here, we sought to investigate how provision of 2′-FL may augment the metabolic output through colonization of *B.p.* MP80 in the murine gut. To understand the strength of the association between 2′-FL and *B.p.* MP80, young adult mice were selected to provide significant colonization resistance. We approached this question by evaluating the gut microbiota and metabolic profiles of mice provided synbiotic treatment containing 2′-FL and *B.p.* MP80 compared with supplementation of either 2′-FL or *B.p.* MP80 alone. This allowed us to gauge the effect of 2′-FL on sustaining *Bifidobacterium* populations in the gut and the corresponding metabolite profiles. Determining how the indigenous microbiota can be modulated by probiotic or synbiotic colonization and the resultant metabolic outputs are critical to understanding how synbiotics may facilitate health outcomes.

## Methods

### Mouse study design

Animals were maintained in accordance with IACUC Protocol 21900 approved by the Institutional Animal Care and Use Committee of University of California, Davis. Male C57BL/6J mice (5–6 weeks old, Jackson Labs) were group housed (3 per cage) and maintained at 22 °C with 12-h light–dark cycle. Before commencing experiments, mice were acclimated for a minimum of 1 week at the facility. Food (5058 Irradiated Pico Mouse Lab Diet) and water were provided ad libitum. 2′-FL was provided in the drinking water as a 10% (w/v) solution. Under anaerobic conditions at 37 °C, *B.p.* MP80 was grown in de Man, Rogosa, and Sharpe media (BD Difco Microbiology, Houston, TX) supplemented with 0.05% w/v L-cysteine (Sigma-Aldrich, St. Louis, MO). *B.p.* MP80 (10^9^ cfu/ml in PBS) or phosphate-buffered saline was administered via oral gavage (100 μl) for 3 days. Within 1 h of the light cycle’s start, fecal samples were collected from individual mice. For validation, three experimental trials were conducted at separate time points. While experimental protocols across trials were preserved, the number of sampling days and final time point did vary between cohorts. For experiments 1 and 2, samples were collected at baseline and days 2, 4, and 6 and a final time point at either day 9 or 10, respectively. For experiment 3, samples were collected at baseline and days 4 and 10. Mice were euthanized via CO_2_ asphyxiation.

### DNA extraction, amplicon sequencing, and bioinformatic processing

DNA was extracted from stool samples (30–100 mg) using the Quick-DNA Fecal/Soil Microbe Miniprep Kit, Catalog No. D6010 (ZYMO, Irvine, CA, USA). Following the manufacturer’s instructions, the extraction protocol included a bead-beating step using a FastPrep-24 Instrument (MP Biomedicals, Santa Ana, CA, USA) for a total of 2 min at 25 °C at a speed of 6.5 m/s. The V4 region of the 16S rRNA gene was amplified in triplicate with barcoded PCR primers F515 (5′-CACGGTCGKCGGCGCCATT-3′) and R806 (5-′GGACTACHVGGGTWTCTAAT-3′) [32] modified to contain an adapter region for sequencing on the Illumina MiSeq platform. Amplicons were verified by gel electrophoresis, combined, purified, and sent to the UC Davis Genome Center for library preparation and high throughput 250-bp paired-end sequencing. Raw sequencing data was demultiplexed before import into QIIME2-2019.10 [33]. The Divisive Amplicon Denoising Algorithm 2 (DADA2) was used for quality filtering and to determine amplicon sequence variants (ASVs) [34]. Reads were trimmed of primers and truncated with a length of 230 bp for forward and 249 bp for reverse. Samples with less than 2000 reads were removed from the ASV table. Taxonomy was assigned using a naïve Bayesian classifier (SILVA 99% 515F/806R) available within QIIME and SILVA ribosomal RNA gene database v138 [35]. Samples were rarified to 3000 sequences. The NCBI BioProject ID for raw 16 s sequencing data is PRJNA725904.

### Statistics (microbial ecosystem)

Microbial community statistical analysis was performed in R (version 4.0.2) [36]. Given the high coefficient of variation of *Bifidobacteriaceae* in synbiotic treated mice, treated mice were classified as highly enriched (HE) if the level of *Bifidobacteriaceae* was greater than the median proportion of *Bifidobacteriaceae* (> 50.5%) and moderately enriched (ME) if the level of *Bifidobacteriaceae* was below the median proportion (< 50.5%). The median proportion of *Bifidobacteriaceae* was calculated from the last available time point (final day for all mice except for one that failed sequencing, in which case day 6 was used) of a larger cohort of mice gavaged with a *Bifidobacterium* species (one arm being *B.p.* MP80) and 2′-FL. This designation was developed to test associations between differential responses (HE vs. ME) to synbiotic treatment.

For each fecal sample, α-diversity was measured with Shannon Index values (vegan::diversity). A linear regression was used to test α-diversity differences between HE and ME bifidobacteria groups among *B.p.* MP80 + 2′-FL treated mice. Included in the linear mixed effects analysis (lme4::lmer) [37] were robust sandwich variance estimates (clubSandwich::vcovCR) [38] and a degrees of freedom Satterthwaite correction (clubSandwich::coef_test). To adjust for lack of independence due to repeated measures, a random intercept model was implemented with mouse ID as the random effect. Selection of which covariates to include in the model was completed using backwards stepwise elimination.

To evaluate microbial community overlap, β-diversity was measured by UniFrac distances (GUniFrac) and visualized using non-metric multidimensional scaling (NMDS) (vegan::metaMDS, *k* = 2) [39]. β-diversity statistical analysis consisted of checking dispersion (vegan::betadisper), a permutational multivariate ANOVA (vegan::adonis2, 999 permutations), and post hoc testing (RVAideMemoire::pairwise.perm.manova, nperm = “500”) [40].

Morisita-Horn dissimilarity index was leveraged to measure the microbial community succession within individual synbiotic treated mice. Values were calculated between baseline and following bacterial gavage (day 4). Identical communities will have a Morisita-Horn index of value of 0 while completely non-overlapping communities will have a value of 1. The generalized linear model analysis (stats::glm, family = binomial(link = probit)) for Morisita-Horn stability (vegan::vegdist, method = “horn”) included robust sandwich variance estimates and a degrees of freedom Satterthwaite correction.

Songbird was employed for differential abundance testing to identify differences in taxa between HE and ME and taxa associated with microbial metabolites. Songbird ranks the log-fold changes between selected features [41]. ASVs were aggregated by bacterial family for Songbird analysis. Songbird analysis used to identify differential taxa between HE and ME categories included samples from SYN treated mice at the final time point and accounted for experimental trial differences. For associations between taxa and metabolites, following the a priori analysis proposal, metabolite levels were dichotomized into high/low based on median level. These high/low values were then used with songbird and the final timepoint 16S rRNA gene sequencing data at the family level to identify bacterial families associated with metabolite levels. In some cases, samples may fail to have a zero abundance of taxa which makes the log ratio impossible to calculate for these samples; should this occur, we will exclude affected samples. Differential abundance log ratios were assessed for normality using a Shapiro–Wilk test to determine if a Student’s *t*-test or Wilcoxon rank sum test should be used. Multiple comparison corrections were applied to account for all analyses using the Benjamini–Hochberg procedure (stats::p.adjust).

A classification tree was generated to differentiate between HE and ME bifidobacteria categorized mice with the minimum split reduced due to a small number of subjects (rpart::rpart, minsplit = 2) [42].

### Metabolomics sample preparation

Colon contents were weighed and combined with 500 μL aliquots of ice-cold PBS. Samples were then vortexed for 2 min, incubated on ice for 5 min and vortexed for 2 additional minutes before centrifugation (6000 × RCF, 15 min, 4° C). Supernatants were transferred to new tubes and pellets were dried in a miVac sample concentrator to determine dry weight. After an additional centrifugation step (14 k RCF, 10 min, 4° C), supernatants were transferred to 3 kDa filters and centrifuged again (14 k RCF, 60 min, 4° C). For each sample, 207 μL of filtrate was transferred to a new tube and combined with 23 μL of internal standard consisting of 4.8 mM DSS-d_6_ containing 0.2% NaN_3_ (to inhibit bacterial growth) in 99.8% D_2_O. The pH of each sample was adjusted to be between 6.7 and 6.9 using NaOH or HCl prior to transfer to 3 mm NMR tubes. Sera were thawed on ice and transferred to 3 kDa filters. After 60 min of centrifugation (4° C, 14,000 × RCF), 207 μL of filtrate was combined with 23 μL of 4.8 mM DSS-d_6_. The pH of each serum sample was adjusted to be between 6.7 and 6.9 using NaOH or HCl. Thawed liver samples were weighed and combined with 900 μL of ice-cold PBS in an MP Bio Lysing matrix D bead beating tube (MP Biomedicals, USA). Samples were homogenized using a FastPrep-24 bead beater (MP Biomedicals, USA) for 60 s at 6 m/s and repeated for a total of 2 min. Liver homogenates were centrifuged for 10 s and cooled on ice for 1 min followed by additional centrifugation for 15 min (14 k RCF, 4° C). Supernatants were transferred to new tubes and spun down for 10 min at 14 k RCF and 4 °C. Supernatants were then transferred to a 3-kD molecular weight filter and centrifuged for 45 min at 14 k RCF and 4 °C. A total of 207 μL of filtrate was transferred to a new tube and combined with 23 μL of 4.8 mM DSS-d_6_, and the pH was adjusted to between 6.7 and 6.9. Brain samples were thawed, weighed, and combined with 550 μL of ice-cold PBS followed by homogenization by bead beating using MB Bio Lysing matrix D bead beating tube (MP Biomedicals, USA) for 1 min at 6 m per second and repeated for a total of 2 min. Samples were spun down for 10 s and incubated on ice for 1 min followed by centrifugation for 15 min at 14 k RCF and 4° C. Supernatants were transferred to new tubes and centrifuged for 10 min at 14 k RCF and 4° C followed by filtering through 3 kD molecular weight filter via centrifugation for 45 min at 14 k RCF and 4 °C. In total, 207 μL of filtrate was combined with 23 μL of 4.8 mM DSS-d_6_ in a new tube and pH was adjusted to between 6.7 and 6.9.

### Acquisition parameters for ^1^H-NMR

^1^H NMR spectra were acquired at 298 K using the NOESY 1H pre-saturation experiment (‘noesypr1d’) on a Bruker Avance 600 MHz NMR spectrometer (Bruker BioSpin, Germany). Data acquisition was achieved with the following parameters: 8 dummy scans and 32 transients over a spectral width of 12 ppm and a total acquisition time of 2.5 s. Water saturation was applied during relaxation delay (2.5 s) and mixing time (100 ms). The resulting spectra were Fourier transformed with zero filling to 128 k data points, and the free induction decays (FIDs) were transformed with an exponential apodization function corresponding to a line broadening of 0.5 Hz. Chenomx NMR Suite v8.4 (Chenomx Inc, Edmonton, Alberta, Canada) was used to manually phase and correct baseline spectra. Each metabolite was assigned manually and quantified using Chenomx Profiler.

### Statistics (metabolites)

All metabolite statistical analyses and graphics were generated using R (v4.0.2). Non-metric multidimensional scaling (NMDS) plots were generated using vegan::metaMDS (k = 2, distance = “euclidian”). Student’s *t*-test was used for group comparisons with homogeneity of variances, else Wilcoxon rank-sum test was used. Kruskal–Wallis rank-sum test was performed for more than two groups followed by Dunn’s test for post hoc evaluation. Homogeneity of variance was tested using Levene’s test (car::leveneTest), and normality was assessed using the Shapiro–Wilk test in addition to observing deviations in the residuals of Quantile–Quantile plots. Corrections were applied for multiple comparisons by Benjamini–Hochberg correction when appropriate. Statistical significance was considered at *α* < 0.05.

## Results

**Fig. 1:**
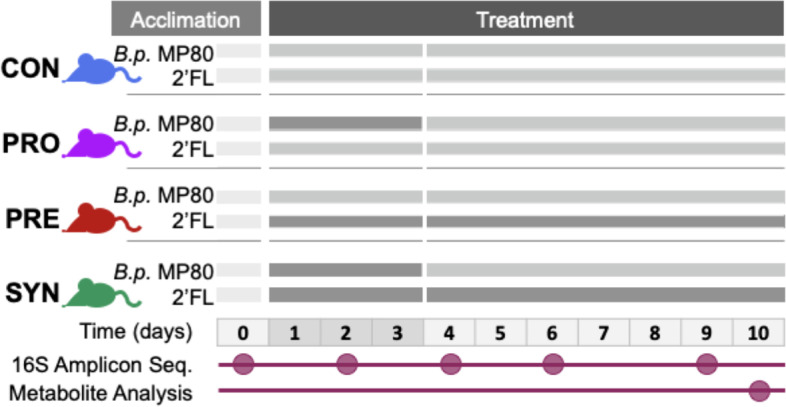
Mouse trial experimental design as a timeline. Treatment groups consisted of control (CON): oral gavage of PBS (days 1–3) and drinking water (days 1–10), probiotic (PRO): oral gavage of *B.p.* MP80 (days 1–3) and drinking water (days 1–10), prebiotic (PRE): oral gavage of PBS (days 1–3) and 2-FL in drinking water (days 1–10), and synbiotic (SYN): oral gavage of *B.p.* MP80 (days 1–3) and 2-FL in drinking water (days 1–10). Fecal samples collected for 16S amplicon sequencing throughout the experiment, samples for metabolite analysis collected during necropsy

To measure changes in metabolism upon supplementation of the bifidobacterial strain, *B.p.* MP80, a total of 39 mice were divided into four groups and provided either the *B.p.* MP80 probiotic (*n* = 9; PRO), 2′-FL as a prebiotic (*n* = 9; PRE), both *B.p.* MP80 and 2′-FL (*n* = 12; SYN), or a PBS control (*n* = 9; CON). The probiotic was provided as an oral gavage each day for 3 days for the PRO and SYN groups, and the prebiotic was provided as a 10% solution of 2′-FL for the PRE and SYN groups every day throughout the experiment (Fig. 1). A total of three experimental trials were conducted for this study with the following sample sizes: trial 1, SYN and CON groups *n* = 3; trial 2, PRO, PRE, SYN, and CON *n* = 3; and trial 3, CON (*n* = 3) and PRO, PRE, and SYN (*n* = 6).

### Persistence of *B.p.* MP80 is permissive when supplemented as a synbiotic but not as a probiotic

**Fig. 2:**
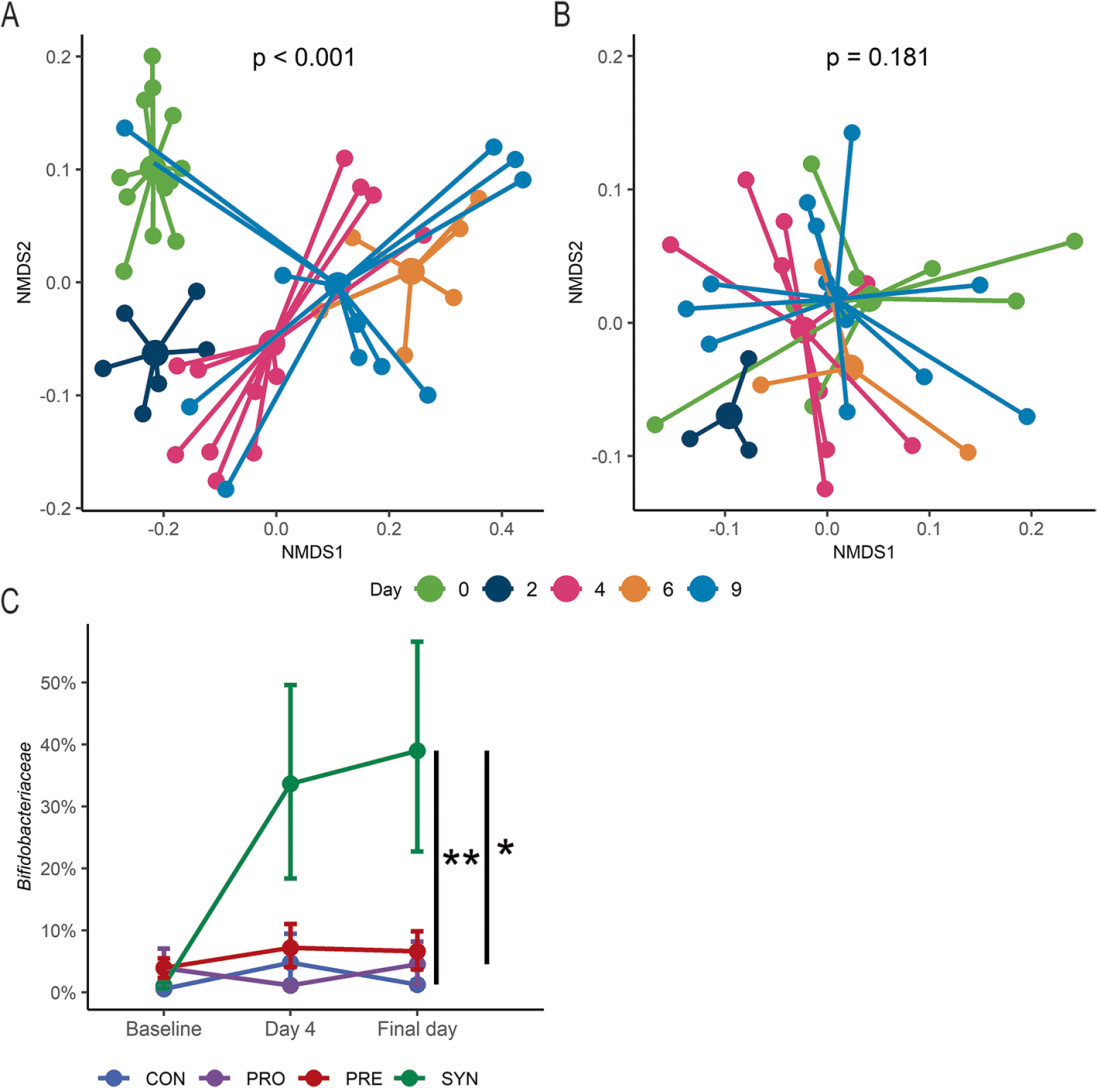
Microbial community structure changes during synbiotic treatment. **A** Microbial community NMDS plot of synbiotic mice (day 0 to final time point) colored by day (*n* = 12); **B** microbial community NMDS plot of probiotic mice (day 0 to final time point) colored by day (*n* = 9); and **C** relative abundance of *Bifidobacteriaceae* in control (*n* = 9), probiotic (*n* = 9), prebiotic (*n* = 9), and synbiotic (*n* = 11) groups from baseline, after oral gavage (day 4) and 1 week following (final day). Final day, while grouped as day 9 for A, consists of day 9 or 10 depending on the experimental trial. NMDS was generated using β-diversity index weighted UniFrac distance in two dimensions. Error bars for relative abundance data is represented as mean and standard error from bootstrapped confidence intervals with 1000 iterations. * *p* < 0.05, ** *p* < 0.01

To assess *B.p.* MP80 persistence in mice, the fecal microbial community structure was evaluated using 16S rRNA amplicon sequencing. Mice in the SYN group had microbial community shifts across each sample day from baseline to the final time point (Fig. 2a, PERMANOVA, *p* < 0.001). Post hoc testing revealed statistical significance between baseline and the subsequent time points (*p* < 0.005). Community structure in the PRO treated mice corresponded to smaller, non-significant shifts during the experiment (Fig. 2b, PERMANOVA, *p* = 0.181). Despite a large variance, persistence of *Bifidobacteriaceae* was observed in the SYN group with significantly higher proportions of *Bifidobacteriaceae* relative to the PRO group at completion of the experiment (Fig. 2c, Wilcoxon rank-sum test, *p* < 0.05). Pairwise comparison of the weighted UniFrac distance revealed that the microbial community structure was statistically different on the final day between each group except for the CON and PRO groups (Table 1, PERMANOVA, *p* < 0.05), suggesting that provision of *B.p.* MP80 alone failed to impact the membership of the microbial community. However, β-diversity at baseline also differed significantly from PRE to CON and SYN treatment groups (Table 1, PERMANOVA, *p* < 0.001) and the three experimental trials (Supplementary Table 1, PERMANOVA, *p* < 0.001).

**Table 1:** P-values of pairwise comparisons of weighted UniFrac measures between treatment groups at the baseline and final time points

	*Baseline*		
	SYN	PRO	PRE
CON	0.567	0.311	**0.040**
PRE	**0.024**	0.40	
PRO	0.311		
	*Final*		
	SYN	PRO	PRE
CON	**0.024**	0.076	**0.006**
PRE	**0.008**	**0.006**	
PRO	**0.009**		

*p-values with statistical significance are indicated in bold

### Gut microbial communities shift dynamically in response to synbiotic treatment

**Fig. 3:**
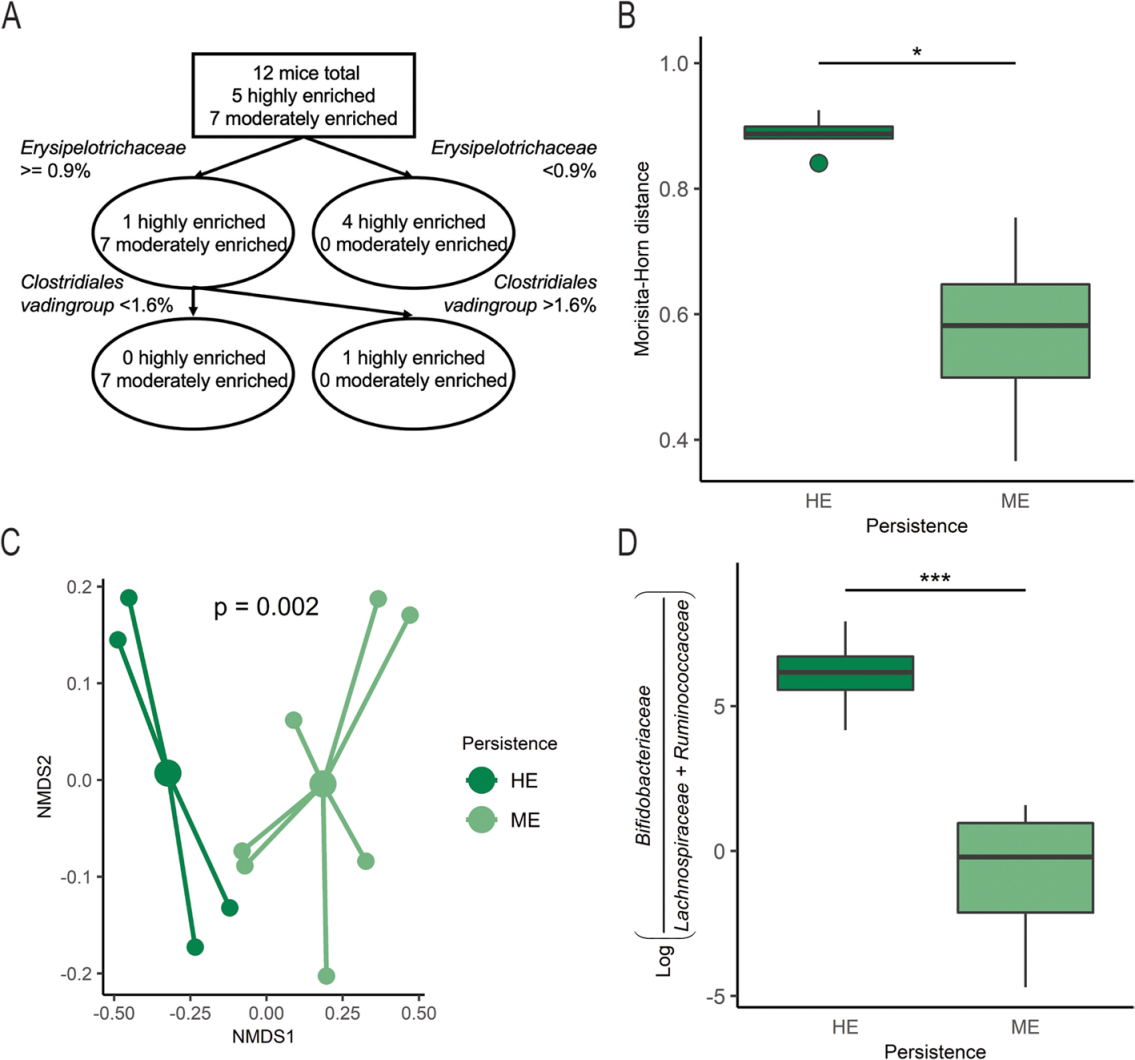
Microbiota community differences between highly enriched (HE) and moderately enriched (ME) bifidobacteria categorizations within synbiotic mice. **A** Classification tree distinguishes mice with HE and ME bifidobacteria persistence based on other microbial taxa; **B** Morisita-Horn distances for synbiotic treated mice from baseline to day following gavage (day 4) grouped as HE (*n* = 5) and ME (*n* = 7) bifidobacteria; **C** microbial community NMDS plot of HE (*n* = 4) and ME (*n* = 7) bifidobacteria groups at the final time point, colored by high and low; **D** log ratio of *Bifidobacteriaceae* relative to *Lachnospiraceae* and *Ruminococcaceae* on the final day of the experiment. Boxplots represent medians and interquartile range (IQR) with whisker end points equal to the maximum and minimum values below or above the median at 1.5 times the IQR. NMDS was generated using β-diversity index weighted UniFrac distance in two dimensions. * p < 0.05, *** p < 0.001

Following completion of bacterial gavage, day 4, we observed a high coefficient of variation in proportions of *Bifidobacteriaceae* in the synbiotic treatment arm (Fig. 2c). We therefore decided to evaluate the microbial diversity within these mice by parsing the group into highly enriched (HE) and moderately enriched (ME) bifidobacterial persistence based on the median relative abundance of *Bifidobacteriaceae* (50.5%) at the final available time point for mice receiving the synbiotic. Microbiota composition was not significantly different at baseline for either α-diversity (Kruskal–Wallis rank-sum test, *p* = 0.223) or β-diversity (weighted UniFrac, *p* = 0.256) suggesting similar initial microbial distribution within and between synbiotic treated mice (Supplementary Fig. 1). Using a linear mixed effects model with sandwich variance, we examined α-diversity over the course of the experiment, accounting for baseline Shannon index, and whether *B.p.* MP80 was supplemented that day (day of gavage). Interestingly, mice with HE bifidobacterial persistence had significantly reduced α-diversity compared to mice with ME bifidobacterial persistence (Table 2). A classification tree explored baseline differences between mice categorized as HE and ME bifidobacteria persistence. The tree identified that 4 of 5 mice with HE bifidobacterial persistence possessed < 0.9% relative abundance *Erysipelotrichaceae* (Fig. 3a). To delineate the stability of the gut microbial communities in mice with HE versus ME persistence, we used a generalized linear regression with sandwich variance to estimate changes in the Morisita-Horn distance from baseline to the day following oral gavage within individual mice, accounting for experimental trial. Mice with HE *Bifidobacteriaceae*, higher *B.p.* MP80 efficiency persistence, had a significantly increased Morisita-Horn distance compared to mice with ME persistence indicating a reduced community stability over the course of *B.p*. MP80 gavage (Table 3, Fig. 3b). At the final time point, 1 week following oral gavage, β-diversity was significantly different in mice with HE compared to ME persistence of *Bifidobacteriaceae* (Fig. 3c, PERMANOVA, *p* < 0.005). Furthermore, differential abundance testing indicated a significantly higher log ratio of *Bifidobacteriaceae* to *Lachnospiraceae* and *Ruminococcaceae* in mice classified with HE bifidobacterial persistence compared to ME persistence (Fig. 3d, t-test, *p* < 0.001). Moreover, the overall median proportions of *Lachnospiraceae* in the HE and ME persistence groups were 0.1% and 12.4% respectively while *Ruminococcaceae* was less than 0.05% and 4.1% respectively (Supplementary Fig. 2), further reflecting the differences in gut microbial representation across synbiotic supplemented mice.

**Table 2:** Linear regression model of Shannon Index (α-diversity) values for SYN treated mice

	Beta coefficients	*t*-statistic	Lower 95% CI	Upper 95% CI	*p*-value*
High Bif. persistence	− 0.578	− 2.74	− 1.07	− 0.09	**0.0271**
Baseline Shannon Index	1.393	3.06	0.27	2.52	**0.0232**
Day of gavage	0.651	4.33	0.32	0.98	** < 0.001**

*p-values with statistical significance are indicated in bold

**Table 3:** Generalized linear regression model of Morisita-Horn (β-diversity) values for SYN treated mice from baseline to day following *B.p.* MP80 gavage

	Beta coefficients	*t*-statistic	Lower 95% CI	Upper 95% CI	*p*-value*
High Bif. persistence	0.930	3.98	0.101	1.778	**0.039**
Experimental trial	− 0.080	− 0.55	− 0.479	0.319	0.608

*p-values with statistical significance are indicated in bold

### Metabolic changes in the gut occur with pre- and syn- but not probiotic treatment

**Fig. 4:**
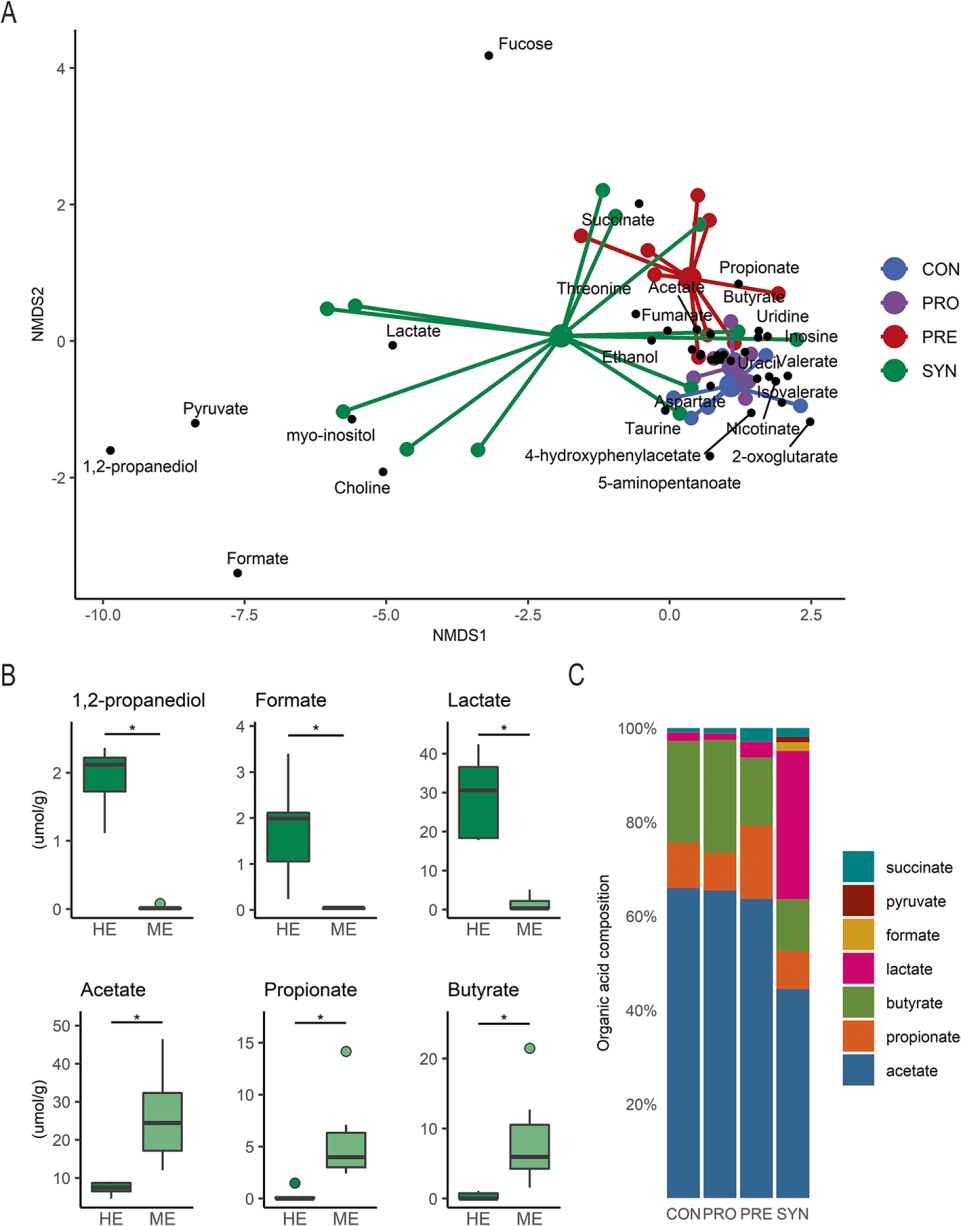
Metabolic profiling of colon contents reveal distinct compositions in synbiotic treated mice with high persistence of bifidobacteria. **A** NMDS plot of mouse colon content metabolome at the final experimental time point; **B** colon content metabolites differ across highly enriched (HE) and moderately enriched (ME) persistence of *Bifidobacteriaceae* by HE (*n* = 4) and ME (*n* = 7); and **C** organic acid composition across treatment groups. NMDS was generated using Euclidian distance in two dimensions. Boxplots represent medians and interquartile range (IQR) with whisker end points equal to the maximum and minimum values below or above the median at 1.5 times the IQR. Organic acid composition was derived by the sum of each acid divided by the total. * p < 0.05

The metabolic output of the gut microbiota inherently depends on the composition of microbes, substrate availability, and their related metabolic activities. Using proton NMR spectroscopy, we interrogated the metabolome of the gut lumen by sampling colon content at the final time point. Non-metric multidimensional scaling (NMDS) revealed considerable overlap of PRO compared with CON group mice suggesting the probiotic alone does not influence microbial metabolites within the gut lumen 1 week after supplementation (Fig. 4a). Prebiotic treated mouse metabolite profiles were distinct from others due to changes driven primarily by monomeric fucose, propionate and succinate reflecting the utilization of the succinate pathway for carbohydrate degradation. Synbiotic treatment resulted in high dispersion in which five mice had distinct gut metabolite profiles that correlated with high lactate, pyruvate, formate, and 1,2-propanediol (1,2-PD), products associated with fucose metabolism in bifidobacteria. Notably, these mice corresponded to those with HE persistence (> 50.5% *Bifidobacteriaceae*), indicating a relationship between these metabolites and the degree of colonization by bifidobacteria. Using the cutoff of 50.5% *Bifidobacteriaceae*, we compared several discriminating metabolites indicated by NMDS. Lactate, formate, and 1,2-PD were all significantly higher in mice with HE persistence, whereas acetate, propionate, and butyrate were significantly lower in mice with HE persistence (Fig. 4b, Wilcoxon rank-sum test, *p* < 0.05). Next, we summed the seven highest concentrated acids and divided by their total to visualize the organic acid composition in the gut and provide an overview of the relative metabolic makeup by treatment (Fig. 4c). Acetate was the dominant metabolite comprising over 60% of the total organic acid content for all except the SYN group, which was approximately 40% of the total. Consistent with the NMDS loadings plot, we observed elevated propionate and succinate in the PRE group relative to the other groups. Moreover, CON and PRO treated mice had similar organic acid profiles to each other which featured higher butyrate proportions compared to PRE and SYN treated mice.

### Metabolic changes in the gut are associated with modulation of the gut microbiota

**Fig. 5:**
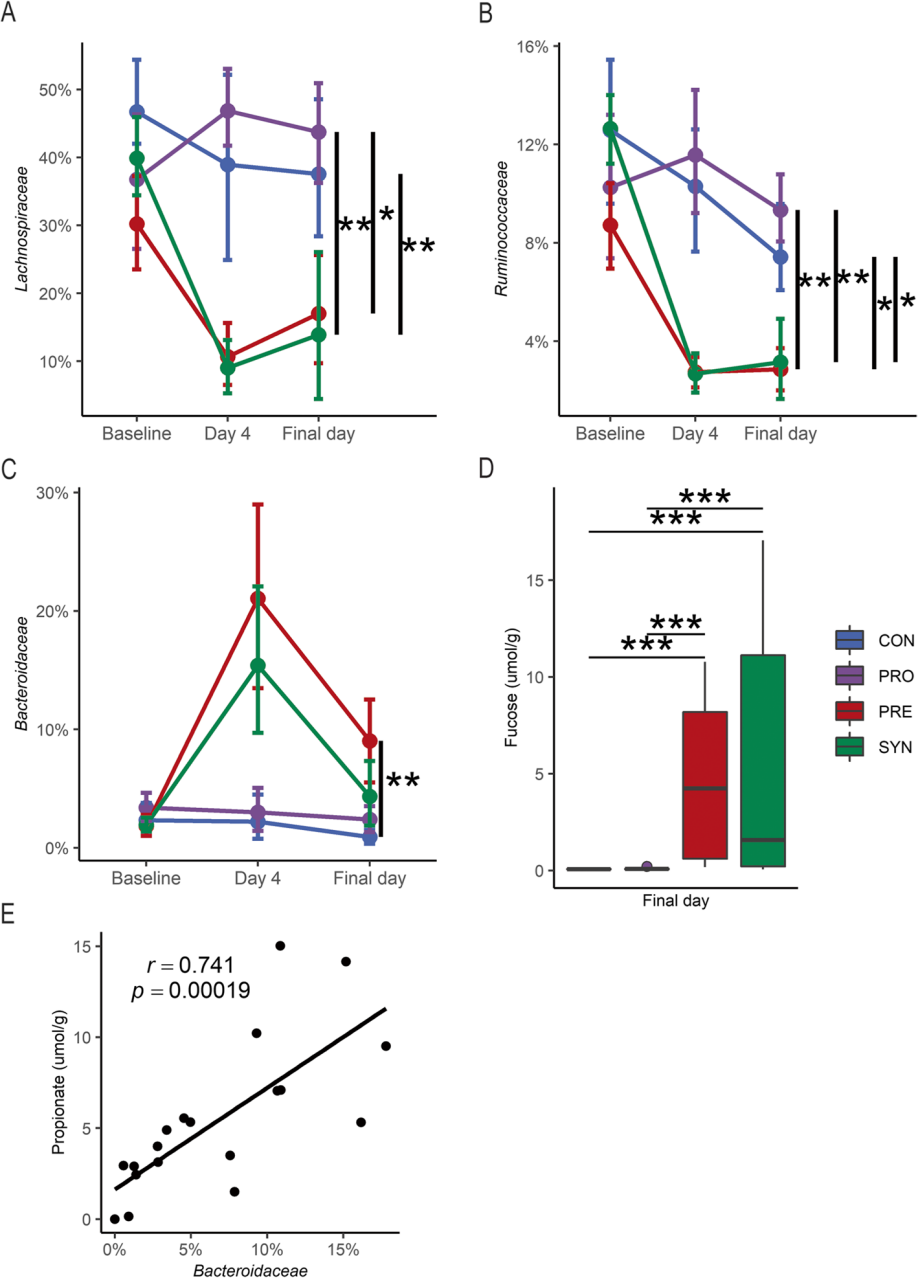
Bacterial relative abundance by treatment group. Relative abundance of **A** *Lachnospiraceae*, **B** *Ruminococcaceae*, and **C** *Bacteroidaceae* in control (*n* = 9), probiotic (*n* = 9), prebiotic (*n* = 9), and synbiotic (*n* = 12) groups from baseline, after oral gavage (day 4) and 1 week following (final day), and **D** free fucose (μmol/g) at the final day for all treatments. Boxplots represent medians and interquartile range (IQR) with whisker end points equal to the maximum and minimum values below or above the median at 1.5 times the IQR. Error bars for relative abundance data is represented as mean and standard error from bootstrapped confidence intervals with 1000 iterations. * *p* < 0.05, ** *p* < 0.01, *** p < 0.001

**Fig. 6:**
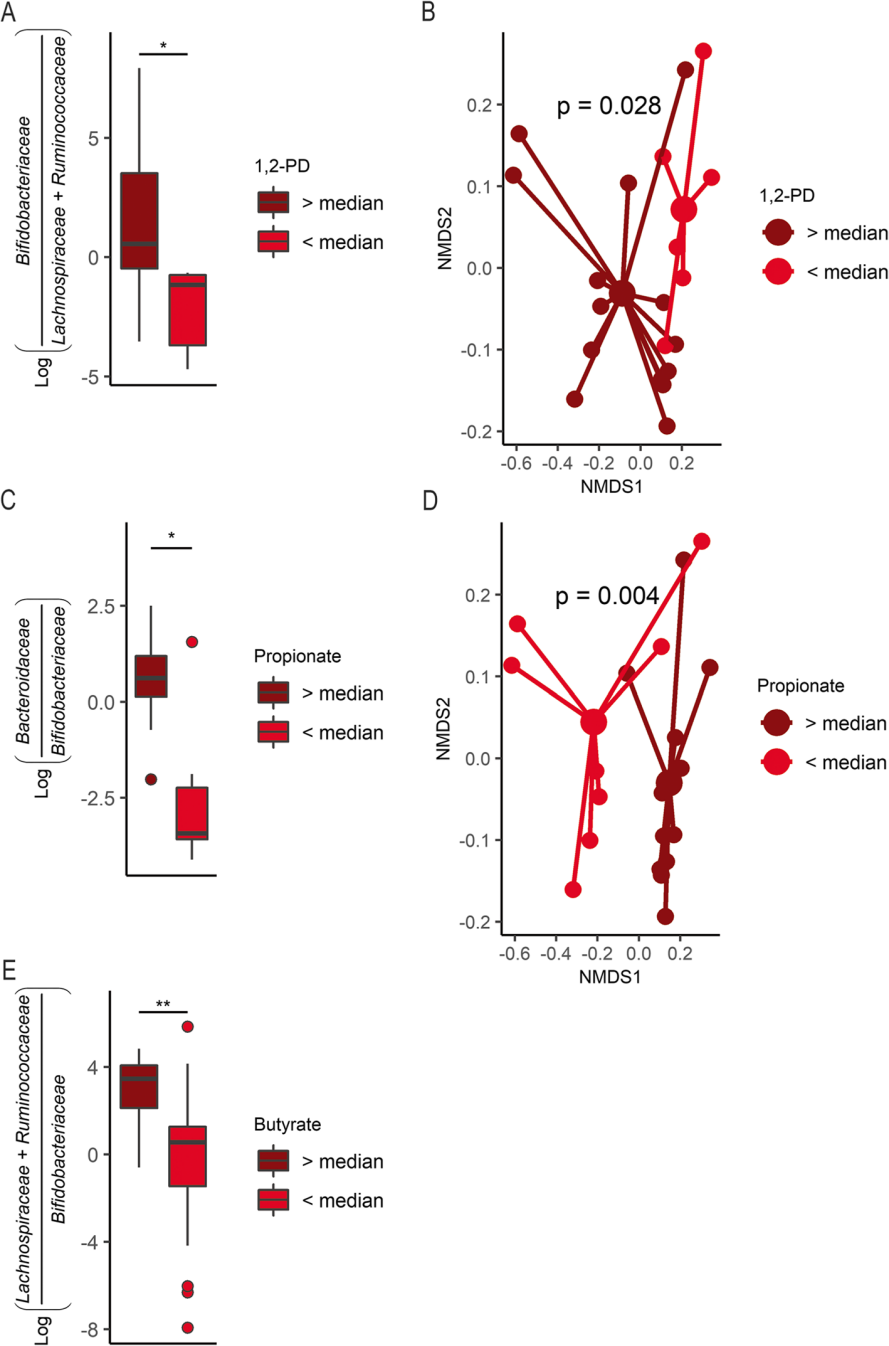
Microbial community differences are associated with distinct metabolite profiles at the final time point. **A** Log ratio of *Bifidobacteriaceae* relative to *Lachnospiraceae* and *Ruminococcaceae* for all 2′-FL mice (PRE + SYN), grouped as either above (*n* = 14) or below (*n* = 6) the median 1,2-propanediol (1,2-PD) concentration and **B** microbial community NMDS plot grouped as above and below the median 1,2-PD concentration; **C** log ratio of *Bacteroidaceae* relative to *Bifidobacteriaceae* for all 2′-FL fed mice (PRE + SYN), grouped as either above (*n* = 12) or below (*n* = 8) the median propionate concentration; **D** microbial community NMDS plot grouped as above and below the median propionate concentration; **E** Log ratio of *Lachnospiraceae* and *Ruminococcaceae* relative to *Bifidobacteriaceae* for all treatments, grouped as either above (*n* = 19) or below (*n* = 19) the median butyrate concentration. Boxplots represent medians and interquartile range (IQR) with whisker end points equal to the maximum and minimum values below or above the median at 1.5 times the IQR. NMDS was generated using β-diversity index weighted UniFrac distance in two dimensions. * p < 0.05, ** p < 0.01

To further assess changes in microbial metabolites related to probiotic, prebiotic, or synbiotic administration, comparisons across treatment arms were performed. Proportions of *Lachnospiraceae* and *Ruminococcaceae* were observed to be significantly higher in CON and PRO groups at the final day of sample collection (Fig. 5a, b, Dunn’s test, *p* < 0.05). The proportion of *Bacteroidaceae* in PRE treated mice was significantly enriched at the final day of sample collection relative to the CON group with concomitantly higher concentrations of free fucose (Fig. 5c, d, Dunn’s test, *p* < 0.01), a phenomenon that has been previously observed during HMO consumption in Bacteroides. Furthermore, a strong correlation between *Bacteroidaceae* and propionate was detected in mice provided 2′-FL (PRE and SYN groups) (Fig. 5e, Pearson’s *r*, *r* = 0.741, *p* < 0.001). To evaluate associations between gut luminal metabolites and corresponding microbiota composition, we used median metabolite concentrations as a cutoff to assess differences in overall microbial community structure and differential abundance of selected bacterial families known to produce those metabolites. First, we examined mice receiving 2′-FL (PRE and SYN groups) in relation to 1,2-PD and propionate as these metabolites are produced by the catabolism of fucose. In mice receiving 2′-FL, the ratio of *Bifidobacteriaceae* to *Lachnospiraceae* and *Ruminococcaceae* was significantly greater with a concentration of 1,2-PD (a fucose metabolite) above the median (Fig. 6a, t-test, *p* < 0.01). Additionally, the median 1,2-PD concentration distinguished microbial communities in 2′-FL fed mice (Fig. 6b, PERMANOVA, *p* < 0.05). Similarly, in mice provided 2′-FL, a significant log ratio increase of *Bacteroidaceae* to *Bifidobacteriaceae* was observed in the high propionate group (Fig. 6c, Wilcoxon rank-sum test, *p* < 0.05), strengthening the link between the co-occurrence of *Bacteroidaceae* with high propionate levels (Fig. 5e). Moreover, colonic propionate at the median cutoff was discriminatory for microbial community structure (Fig. 6d, PERMANOVA, *p* < 0.005). For all mice, higher butyrate concentrations had a significantly higher log ratio of *Lachnospiraceae* and *Ruminococcaceae* relative to *Bifidobacteriaceae* (Fig. 6e, Wilcoxon rank-sum test, *p* < 0.01). However, differences in the microbial community composition split at the median gut butyrate concentration could not be tested by PERMANOVA due to unequal group dispersions.

### Synbiotic treatment affects systemic metabolism

**Fig. 7:**
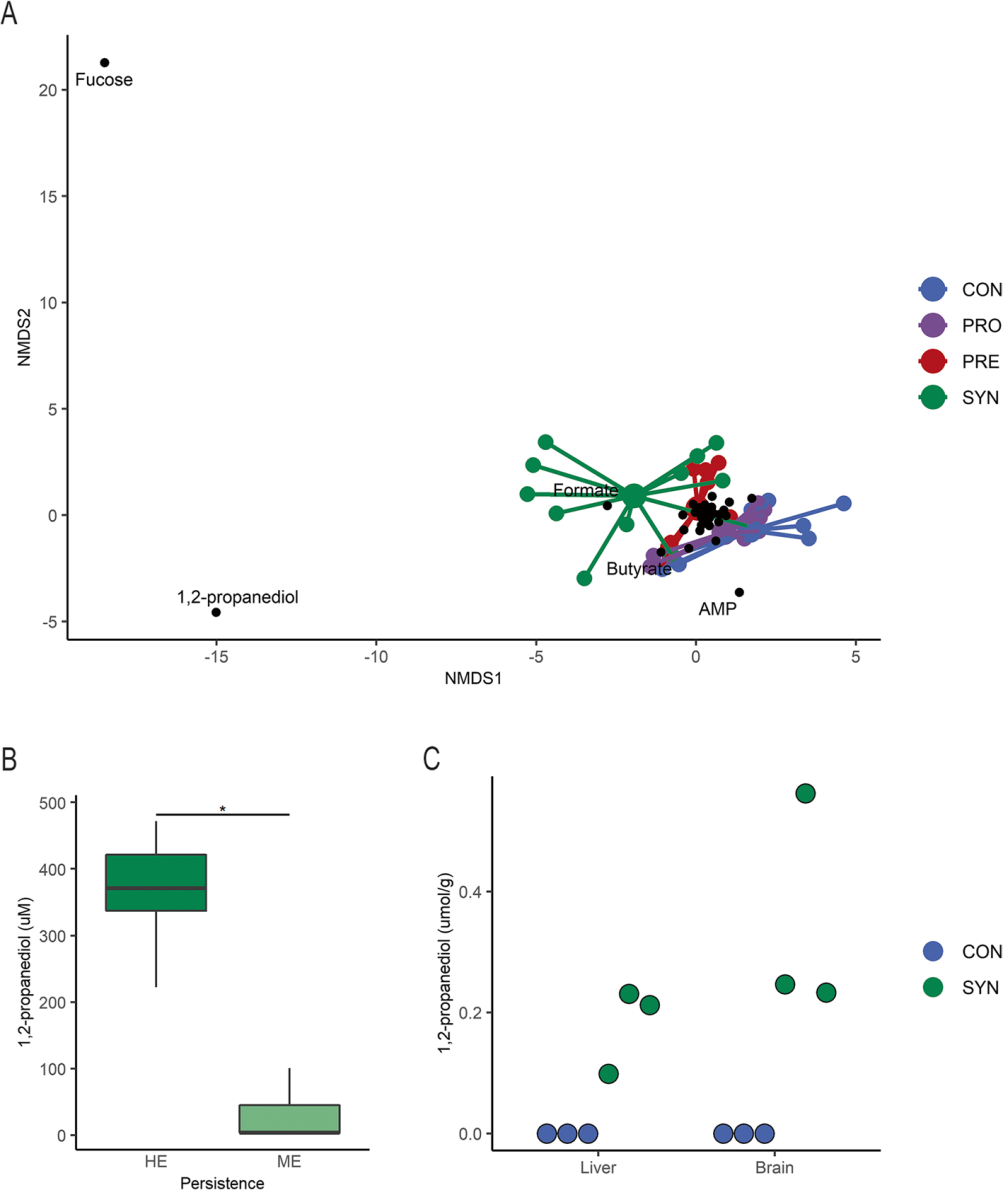
Systemic metabolome changes associated with synbiotic treatment. **A** NMDS plot of mouse serum metabolome at the final experimental time point, with metabolites highlighted that drive differentiation of samples, colored by treatment group; **B** serum 1,2-propanediol (μmol/g) by highly enriched (HE) and moderately enriched (ME) bifidobacteria categorization of synbiotic treated mice; and **C** liver and brain 1,2-propanediol for control (*n* = 3) and synbiotic (*n* = 3) mice from experimental trial one. NMDS was generated using Euclidian distance in two dimensions. Boxplots represent medians and interquartile range (IQR) with whisker end points equal to the maximum and minimum values below or above the median at 1.5 times the IQR. * p < 0.05

Serum obtained on the final day was analyzed for metabolomics to discern changes in systemic metabolites related to treatment. Ordination by NMDS showed similar results as observed with colon content metabolites, overlap of CON and PRO groups followed by some separation with PRE treatment and diffuse SYN group dispersion (Fig. 7a). Notably, the five mice exhibiting the greatest separation across the first NMDS axis from the control groups were identified as those with HE persistence (> 50.5% *Bifidobacteriaceae*) and distinct gut metabolite profiles (Fig. 4a). Metabolic features related to separation in serum were predominately fucose, formate, and 1,2-PD. The higher concentrations of the fucose metabolite 1,2-PD found in the colon contents of mice with high bifidobacterial persistence were also significantly higher in the serum of these same animals (Fig. 7b, t-test, *p* < 0.05) suggesting increased absorption across the gut epithelium. Lastly, we examined the liver and brain to determine if the perfusion of blood with enriched microbial metabolites equilibrated with these organs. Notably, only synbiotic treated mice showed detectable concentrations of 1,2-PD in liver and brain samples with none detected in the control animals (Fig. 7c). Together, only persistent colonization corresponded to an increase in the microbial fermentation products 1,2-PD observed in circulation. The presence of 1,2-PD in liver and brain further indicates a systemic distribution of metabolites that occurs during synbiotic treatment.

## Discussion

Here, we demonstrate that the synbiotic provision of *B.p.* MP80 and 2′-FL in the conventionally colonized murine gut is capable of modulating the output of microbial metabolites both locally and systemically. Bifidobacterial-associated HMO fermentation products scaled with the enrichment of *B.p.* MP80 in synbiotic treated mice, whereas gut metabolite profiles of mice provided only the probiotic were indistinguishable from mice receiving neither the probiotic nor the prebiotic at the conclusion of this study. Our previous work established that 2′-FL is sufficient to facilitate persistence of *B.p.* MP80 in a competitive environment [28]. Concordant with that study, we observed synbiotic treatment, rather than the probiotic alone, resulted in persistent colonization of *Bifidobacterium*, albeit at variable levels of enrichment. In the current study, metabolite profiling of the gut lumen, liver, and brain provided a means to interrogate the effect of synbiotic treatment on host-microbe co-metabolism. The inability of a novel intestinal microbe to compete with the indigenous microbial community is well established with studies indicating the degree of individual permissiveness to an invading microbe is contingent on the baseline microbial composition [18, 19, 43]. However, in this study, no baseline microbial community distinction (α-diversity or β-diversity) was found between groups with HE and ME persistence of *Bifidobacteriaceae*. Stratification into high and low proportions of bifidobacteria were distinct throughout the experiment by α-diversity, exemplifying the diversity-invasion effect where survival of an invader, *B.p.* MP80, is inversely correlated to species richness and evenness [44, 45]. A classification tree identified very low baseline *Erysipelotrichaceae* relative abundance being associated with HE bifidobacterial persistence. Currently, an unknown aspect of *Erysipelotrichaceae* appears to be preventing *B.p.* MP80 from effectively exploiting the 2′-FL nutrient niche and outcompeting endogenous microbes. Further investigation of *Erysipelotrichaceae* and colonization resistance to synbiotic treatment is required. At the final time point, the microbial community structure (β-diversity) was distinct within the SYN group, resulting from the division of HE and ME *Bifidobacteriaceae* persistence.

In infants, the functional capacity to catabolize HMOs is associated with high levels of *Bifidobacterium* and their metabolites [4, 17, 30, 46, 47]. In our mouse model, a persistent, predominant *B.p.* MP80 population generated discrete metabolic profiles defined by elevated lactate, formate and 1,2-PD in the colon. Additionally, this metabolic capacity aligns with prior in vitro analysis of *B.p.* MP80 2′-FL catabolism, leading us to conclude that the degree of colonization in vivo is commensurate with the enrichment of 2′-FL metabolites produced by *Bifidobacterium* including lactate and 1,2-PD [29]. These products are found in considerably lower quantities in the adult gut due to either a reduced capacity to produce these metabolites and/or their utilization by other microbial inhabitants [48]. As such, a lower diversity ecosystem dominated by bifidobacteria as observed in the mice with high persistence of bifidobacteria has greater potential to accumulate these products.

Diet-driven microbial metabolic effects have been widely studied in humans and animal models due to associated health benefits [49–51]. Here, we identified gut microbiota compositions are distinguished by their metabolic output (butyrate, propionate, and 1,2-PD). Butyrate-producing bacteria create a functional cohort where the two most abundant groups include *Eubacterium rectale/Roseburia spp. (Lachnospiraceae)* and *Faecalibacterium prausnitzii (Ruminococcaceae)* [52]. As butyrate production is due to the breakdown of complex polysaccharides that reach the colon, it is appropriate that we found butyrate concentrations associated with enrichment of *Lachnospiraceae* and *Ruminococcaceae* families in mice where chow was the predominant fiber source. However, in mice with 2′-FL supplementation, butyrate concentrations were proportionally lower, coinciding with a previous report in which 2′-FL supplementation in mice was associated with reduced butyrate [53]. Following baseline, we observed provision of 2′-FL in the PRE and SYN groups corresponded with enrichment of *Bacteroidaceae* in the murine gut at each sampling period. *Bacteroides* species typically possess several polysaccharide utilization loci in their genomes that enable cleavage of a variety of glycosidic linkages including HMOs [54]. Metabolically, *Bacteroides* is a primary producer of propionate in the gut microbiome via the succinate pathway [55, 56]. Therefore, in cases where 2′-FL provision resulted in high propionate, indigenous *Bacteroidaceae* likely outcompeted the autochthonous microbial community in prebiotic fed mice and *B.p.* MP80 in those receiving the synbiotic. This model draws parallels with previous reports that describe the high prevalence of *Bacteroides* in the gut microbiota of breastfed infants which likely arises from utilization of HMOs [57]. Competition between *Bacteroides* and *Bifidobacterium* for the HMO nutrient niche [58] is recapitulated in this model, as evidenced by the greater enrichment of *Bacteroidaceae* in the mice receiving only 2′-FL (PRE) relative to those provided the synbiotic which had higher proportions of *Bifidobacteriaceae*. Additionally, mice with high concentrations of 1,2-PD in the gut were enriched in *Bifidobacteriaceae*. The propanediol pathway, common to *Bifidobacterium*, characteristically produces 1,2-PD from the metabolism of fucose which is found to be elevated in infants enriched with *Bifidobacteriaceae* [29, 59–61]. In our model, high concentrations of 1,2-PD were observed only in mice with high *Bifidobacteriaceae* suggesting a related source of this metabolite.

Some microbial metabolites produced in the intestine can be absorbed across the gut epithelium into systemic circulation. We found that each treatment group exhibited a similar pattern of serum and colon content metabolite profiles. Notably, significantly higher concentrations of 1,2-PD in serum were observed in mice with high persistence of *Bifidobacteriaceae*, suggesting that in this model probiotic persistence (and hence synbiotic supplementation) is necessary to produce metabolites at a sufficient concentration to become systemically enriched. Evidence of this relationship has been shown in human adults during which the provision of *Bifidobacterium animalis* subsp. *lactis* (*B. lactis*) and fructooligosaccharides had a more pronounced effect on serum metabolites compared to *B. lactis* alone [62]. Moreover, the absorption of microbial metabolites into circulation has the potential to interact with peripheral tissues and influence their metabolism. Metabolic profiles in these tissues were similar across groups with the exception of 1,2-PD which was only detected in the liver and brain of SYN treated mice. Based on these data, we conclude that the high concentration of 1,2-PD in the gut generated by microbial fermentation of fucose was absorbed via portal circulation passing through the liver and subsequently reached other peripheral tissues including the brain. Prior work using ^13^C-labeled 2′-FL orally administered to mice showed that ^13^C enrichment occurred in tissues including liver and brain [63]. Moreover, in their study, 2′-FL administered to germ-free mice failed to retain the ^13^C label in their tissues indicating that the gut microbiota is fundamental to enrichment of 2′-FL. Additionally, intravenous administration resulted in ^13^C being excreted in urine further implicating that microbial metabolism is a precursor to tissue incorporation. Our work provides evidence that fermentation of 2′-FL by gut microbes produce metabolites that enter circulation. It is likely that these metabolites play an important role in metabolism in peripheral tissues, although this has not been shown to date. This is potentially important considering the assembly of the gut microbiota in early life could tune host metabolic processes that impact cognitive and metabolic development. Understanding the contribution of microbial metabolites at this stage of life will be instrumental in the development and use of biologics to confer well-being throughout the lifespan.

## Conclusions

In summary, we found that introducing *B.p.* MP80 into the colonized murine gut environment requires concomitant provision of a nutrient niche (2′-FL) to modulate metabolism at the local and systemic level. Without this advantage, colonization resistance cannot be overcome as the community structure reconfigures to the pre-treatment condition following probiotic inoculation. Additionally, this reinforces the finding that HMOs act as a privileged nutrient resource for a competitive population of *Bifidobacterium*, although the permissiveness to colonization does have variability. Moreover, enrichment of microbial metabolites is dependent on a high-degree of persistent inhabitance of *B.p.* MP80 and is reflected by bifidobacterial products of 2′-FL catabolism throughout the host organism.

Our study does have limitations; although mice were ordered through the same facility, the baseline microbial community structure was distinct between trials. This limitation directly affected differential abundance testing where ASVs were grouped by bacterial family for data processing. While this reduced analysis granularity, the fact that differential abundance at the bacterial family level was statistically significant demonstrates how strongly associated diet and microbial communities were within this trial. Additionally, the number of mice in each group were not evenly distributed among experimental trials that may contribute to microbial community differences between treatments at baseline. The established microbial community of the mouse gut does not perfectly exemplify the human gut microbiota nor identically capture the competition for resources and available physical niches. This model assessed how infant-borne *Bifidobacterium* strains are competitive, persistent, and metabolically active when a privileged nutrient source is provided, even in a non-indigenous environment. Developing an ecological framework with the use of synbiotics is crucial for discovering the underlying mechanisms of synbiotic-associated health outcomes. Overall, such findings will aid in pinpointing synbiotic pairings that possess a higher likelihood of conferring health benefits to the host.

### Supplementary Information

**Additional file 1:** **Supplementary Table 1.** Pairwise comparisons* of weighted UniFrac measures between experimental trials at baseline. *Comparisons were evaluated using PERMANOVA and FDR adjustment. P-values with statistical significance are denoted in bold. **Supplementary Figure 1.** Baseline differences not significant between high and low bifidobacteria categorizations of synbiotic treated mice. (A) Shannon α-diversity index values at baseline for synbiotic treated mice grouped as highly enriched (HE) (*n* = 4) and moderately enriched (ME) (*n* = 7) bifidobacteria persistence; and (B) NMDS plot of β-diversity index weighted UniFrac for high and low bifidobacteria groups at baseline. Boxplots represent medians and interquartile range (IQR) with whisker end points equal to the maximum and minimum values below or above the median at 1.5 times the IQR. **Supplementary Figure 2.** Relative abundance of *Lachnospiraceae* and *Ruminococcaceae* at the final time point for 2’-FL treated mice grouped as highly enriched (HE) (*n* = 4) and moderately enriched (ME) (*n* = 7) *Bifidobacteriaceae* based on median relative abundance (50.5%). Boxplots represent medians and interquartile range (IQR) with whisker end points equal to the maximum and minimum values below or above the median at 1.5 times the IQR.

## Data Availability

All data generated or analyzed during this study are included in this published article [and its supplementary information files]. Datasets and scripts used for reproducing the analysis are available at https://github.com/jalarke/bifidobacterium_mouse_persist.

## Abbreviations

HMO: Human milk oligosaccharide
2′-FL: 2′-Fucosyllactose
*B.p.* MP80: *Bifidobacterium* *pseudocatenulatum* MP80
PRO: Group receiving *B.p.* MP80 only
PRE: Group receiving 2′-FL only
SYN: Group receiving *B.p.* MP80 and 2′-FL
CON: Control group
HE: Highly enriched
ME: Moderately enriched
NMDS: Non-metric multidimensional scaling
1,2-PD: 1,2-Propanediol
*B. ** lactis*: *Bifidobacterium* *animalis* subsp. *lactis*
cfu: Colony forming units

## References

[1] György P, Norris RF, Rose CS (1954). Bifidus factor. I. A variant of *Lactobacillus bifidus* requiring a special growth factor. Arch Biochem Biophys..

[2] Davis JCC (2016). Identification of Oligosaccharides in feces of breast-fed infants and their correlation with the gut microbial community. Mol Cell Proteomics.

[3] Totten SM (2012). Comprehensive profiles of human milk oligosaccharides yield highly sensitive and specific markers for determining secretor status in lactating mothers. J Proteome Res.

[4] Sakanaka M (2019). Evolutionary adaptation in fucosyllactose uptake systems supports bifidobacteria-infant symbiosis. Sci Adv..

[5] Lawson MAE (2020). Breast milk-derived human milk oligosaccharides promote *Bifidobacterium* interactions within a single ecosystem. ISME J.

[6] Vatanen T (2019). Genomic variation and strain-specific functional adaptation in the human gut microbiome during early life. Nat Microbiol.

[7] Karav S, Casaburi G, Frese SA (2018). Reduced colonic mucin degradation in breastfed infants colonized by *Bifidobacterium **longum* subsp. *infantis* EVC001. FEBS Open Bio.

[8] Casaburi G (2019). Early-life gut microbiome modulation reduces the abundance of antibiotic-resistant bacteria. Antimicrob Resist Infect Control.

[9] Matsuki T (2016). A key genetic factor for fucosyllactose utilization affects infant gut microbiota development. Nat Commun.

[10] Henrick BM (2019). Colonization by B. infantis EVC001 modulates enteric inflammation in exclusively breastfed infants. Pediatr Res.

[11] Lewis ZT (2015). Maternal fucosyltransferase 2 status affects the gut bifidobacterial communities of breastfed infants. Microbiome.

[12] Vatanen T (2016). Variation in microbiome LPS immunogenicity contributes to autoimmunity in humans. Cell.

[13] Kalliomäki M, Carmen Collado M, Salminen S, Isolauri E (2008). Early differences in fecal microbiota composition in children may predict overweight. Am J Clin Nutr.

[14] Stanislawski MA, et al. Gut microbiota in the first 2 years of life and the association with body mass index at age 12 in a Norwegian birth cohort. mBio. 2018;9:e01751–18. 10.1128/mBio.01751-18 PMC6199494 30352933

[15] Huda MN (2019). *Bifidobacterium* abundance in early infancy and vaccine response at 2 years of age. Pediatrics.

[16] Taft DH, et al. Bifidobacterial dominance of the gut in early life and acquisition of antimicrobial resistance. mSphere. 2018;3:e00441–18. 10.1128/mSphere.00441-18 PMC6158511 30258040

[17] Nguyen M (2021). Impact of probiotic *B. **infantis* EVC001 feeding in premature infants on the gut microbiome, nosocomially acquired antibiotic resistance, and enteric inflammation. Front Pediatr.

[18] Zmora N (2018). Personalized gut mucosal colonization resistance to empiric probiotics is associated with unique host and microbiome features. Cell.

[19] Maldonado-Gómez MX (2016). Stable engraftment of *Bifidobacterium **longum* AH1206 in the human gut depends on individualized features of the resident microbiome. Cell Host Microbe.

[20] Martínez I. et al. Experimental evaluation of the importance of colonization history in early-life gut microbiota assembly. eLife. 2018;7:e36521. 10.7554/eLife.36521 PMC6143339 30226190

[21] Benson AK (2010). Individuality in gut microbiota composition is a complex polygenic trait shaped by multiple environmental and host genetic factors. Proc Natl Acad Sci.

[22] Carmody RN (2015). Diet dominates host genotype in shaping the murine gut microbiota. Cell Host Microbe.

[23] Suez J, Zmora N, Segal E, Elinav E (2019). The pros, cons, and many unknowns of probiotics. Nat Med.

[24] Swanson KS (2020). The International Scientific Association for Probiotics and Prebiotics (ISAPP) consensus statement on the definition and scope of synbiotics. Nat Rev Gastroenterol Hepatol.

[25] Bertuzzo E (2011). Spatial effects on species persistence and implications for biodiversity. Proc Natl Acad Sci.

[26] Walter J, Maldonado-Gómez MX, Martínez I (2018). To engraft or not to engraft: an ecological framework for gut microbiome modulation with live microbes. Curr Opin Biotechnol.

[27] Zhang C (2016). Ecological robustness of the gut microbiota in response to ingestion of transient food-borne microbes. ISME J.

[28] Heiss BE (2021). *Bifidobacterium* catabolism of human milk oligosaccharides overrides endogenous competitive exclusion driving colonization and protection. Gut Microbes.

[29] Shani G (2022). Fucosylated human milk oligosaccharide foraging within the species *Bifidobacterium **pseudocatenulatum* is driven by glycosyl hydrolase content and specificity. Appl Environ Microbiol.

[30] Frese SA, et al. Persistence of supplemented *Bifidobacterium longum* subsp. *infantis* EVC001 in breastfed infants. mSphere. 2017;2:e00501–17. 10.1128/mSphere.00501-17 PMC5717325 29242832

[31] O’Brien CE, et al. Early probiotic supplementation with *B. infantis* in breastfed infants leads to persistent colonization at 1 year. Pediatr Res. 2021;91:627–636. 10.1038/s41390-020-01350-0 PMC8460680 33762689

[32] Caporaso JG (2011). Global patterns of 16S rRNA diversity at a depth of millions of sequences per sample. Proc Natl Acad Sci.

[33] Bolyen E (2019). Reproducible, interactive, scalable and extensible microbiome data science using QIIME 2. Nat Biotechnol.

[34] Callahan BJ (2016). DADA2: high-resolution sample inference from Illumina amplicon data. Nat Methods.

[35] Quast C (2012). The SILVA ribosomal RNA gene database project: improved data processing and web-based tools. Nucleic Acids Res.

[36] R Core Team (2020). R: a language and environment for statistical computing.

[37] Bates D, Mächler M, Bolker B, Walker S (2015). Fitting linear mixed-effects models using lme4. J Stat Soft.

[38] Pustejovsky J. clubSandwich: cluster-robust (Sandwich) variance estimators with small-sample corrections. R package. 2021.

[39] Oksanen J, et al. vegan: community ecology package. R package version 2.5–7. R package version 2.5–7. 2020.

[40] Hervé M. RVAideMemoire: testing and plotting procedures for biostatistics. R package version 0.9–79. 2021.

[41] Morton JT (2019). Establishing microbial composition measurement standards with reference frames. Nat Commun.

[42] Therneau TM, & Atkinson EJ. RPART: recursive partitioning and regression trees. R package version 4.1–15. 2019. https://CRAN.R-project.org/package=rpart.

[43] Davis LMG, Martínez I, Walter J, Goin C, Hutkins RW (2011). Barcoded pyrosequencing reveals that consumption of galactooligosaccharides results in a highly specific bifidogenic response in humans. PLoS ONE.

[44] Mallon CA, van Elsas JD, Salles JF (2015). Microbial invasions: the process, patterns, and mechanisms. Trends Microbiol.

[45] van Elsas JD (2012). Microbial diversity determines the invasion of soil by a bacterial pathogen. Proc Natl Acad Sci.

[46] Chow J (2014). Fecal metabolomics of healthy breast-fed versus formula-fed infants before and during in vitro batch culture fermentation. J Proteome Res.

[47] He X (2019). Fecal microbiome and metabolome of infants fed bovine MFGM supplemented formula or standard formula with breast-fed infants as reference: a randomized controlled trial. Sci Rep.

[48] Flint HJ, Duncan SH, Scott KP, Louis P (2015). Links between diet, gut microbiota composition and gut metabolism. Proc Nutr Soc.

[49] So D (2018). Dietary fiber intervention on gut microbiota composition in healthy adults: a systematic review and meta-analysis. Am J Clin Nutr.

[50] Krumbeck JA, Walter J, Hutkins RW (2018). Synbiotics for improved human health: recent developments, challenges, and opportunities. Ann Rev Food Sci Technol.

[51] Krumbeck JA, Maldonado-Gomez MX, Ramer-Tait AE, Hutkins RW (2016). Prebiotics and synbiotics: dietary strategies for improving gut health. Curr Opin Gastroenterol.

[52] Louis P, Flint HJ (2009). Diversity, metabolism and microbial ecology of butyrate-producing bacteria from the human large intestine. FEMS Microbiol Lett.

[53] Lee S (2020). 2′-Fucosyllactose supplementation improves gut-brain signaling and diet-induced obese phenotype and changes the gut microbiota in high fat-fed mice. Nutrients.

[54] Marcobal A, Sonnenburg JL (2012). Human milk oligosaccharide consumption by intestinal microbiota. Clin Microbiol Infect.

[55] Salonen A (2014). Impact of diet and individual variation on intestinal microbiota composition and fermentation products in obese men. ISME J.

[56] Louis P, Flint HJ (2017). Formation of propionate and butyrate by the human colonic microbiota. Environ Microbiol.

[57] Rotimi VO, Duerden BI (1981). *Bacteroides* species in the normal neonatal faecal flora. J Hyg.

[58] Marcobal A (2011). Bacteroides in the infant gut consume milk oligosaccharides via mucus-utilization pathways. Cell Host Microbe.

[59] He X, et al. Metabolic phenotype of breast-fed infants, and infants fed standard formula or bovine MFGM supplemented formula: a randomized controlled trial. Sci Rep. 2019;9:339. 10.1038/s41598-018-36292-5 PMC6344597 30674917

[60] Lee H (2021). Milk fat globule membrane as a modulator of infant metabolism and gut microbiota: a formula supplement narrowing the metabolic differences between breastfed and formula-fed infants. Mol Nutr Food Res.

[61] Larke J (2022). Premature infant fecal metabolite profiles are modulated in a probiotic specific manner. J Pediatr Gastroenterol Nutr.

[62] Crovesy L, El-Bacha T, Rosado EL (2021). Modulation of the gut microbiota by probiotics and synbiotics is associated with changes in serum metabolite profile related to a decrease in inflammation and overall benefits to metabolic health: a double-blind randomized controlled clinical trial in women with obesity. Food Funct.

[63] Kuntz S (2019). Metabolic fate and distribution of 2´-fucosyllactose: direct influence on gut microbial activity but not on brain. Mol Nutr Food Res.

