# Supplementary material for: Milk oligosaccharide-driven persistence of Bifidobacterium pseudocatenulatum modulates local and systemic microbial metabolites upon synbiotic treatment in conventionally colonized mice
Source: Microbiome. 2023 Aug 28;11:194. doi: 10.1186/s40168-023-01624-9 (PMC10463478; doi:10.1186/s40168-023-01624-9)
Supplement: Supplementary file 2 — Additional file 1: Supplementary Table 1. Pairwise comparisons* of weighted UniFrac measures between experimental trials at baseline. *Comparisons were evaluated using PERMANOVA and FDR adjustment. P-values with statistical significance are denoted in bold. Supplementary Figure 1. Baseline differences not significant between high and low bifidobacteria categorizations of synbiotic treated mice. (A) Shannon α-diversity index values at baseline for synbiotic treated mice grouped as highly enriched (HE) (n = 4) and moderately enriched (ME) (n = 7) bifidobacteria persistence; and (B) NMDS plot of β-diversity index weighted UniFrac for high and low bifidobacteria groups at baseline. Boxplots represent medians and interquartile range (IQR) with whisker end points equal to the maximum and minimum values below or above the median at 1.5 times the IQR. Supplementary Figure 2. Relative abundance of Lachnospiraceae and Ruminococcaceae at the final time point for 2’-FL treated mice grouped as highly enriched (HE) (n = 4) and moderately enriched (ME) (n = 7) Bifidobacteriaceae based on median relative abundance (50.5%). Boxplots represent medians and interquartile range (IQR) with whisker end points equal to the maximum and minimum values below or above the median at 1.5 times the IQR. [file 40168_2023_1624_MOESM1_ESM.pdf]

Supplementary Table and Figures

Milk oligosaccharide driven persistence of *Bifidobacterium pseudocatenulatum* modulates local and systemic microbial metabolites upon synbiotic treatment in conventionally colonized mice

Jules A. Larke[1*], Britta E. Heiss[2*], Amy M. Ehrlich[3], Diana H. Taft[2], Helen E. Raybould[3], and David A. Mills[2], Carolyn M. Slupsky[1,2]

[1]Department of Nutrition [2]Department of Food Science and Technology, [3]Department of Anatomy, Physiology, and Cell Biology, School of Veterinary Medicine, University of California, Davis, Davis, CA, USA

Supplementary Table 1. Pairwise comparisons* of weighted UniFrac measures between experimental trials at baseline.

	First Exp. Trial	Second Exp. Trial
Second Experimental Trial	**0.021**	
Third Experimental Trial	**0.021**	0.148

*Comparisons were evaluated using PERMANOVA and FDR adjustment.
P-values with statistical significance are denoted in bold.

A B

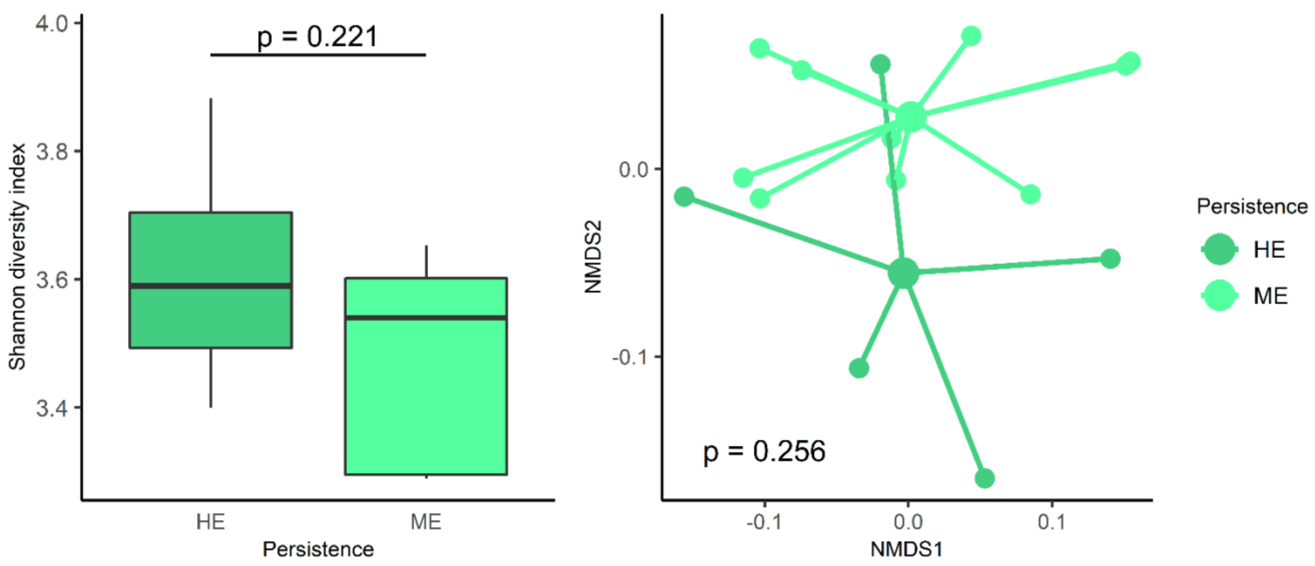

Supplementary Figure 1. Baseline differences not significant between high and low bifidobacteria categorizations of synbiotic treated mice. (A) Shannon α-diversity index values at baseline for synbiotic treated mice grouped as highly enriched (HE) (n = 4) and moderately enriched (ME) (n = 7) bifidobacteria persistence; and (B) NMDS plot of β-diversity index weighted unifrac for high and low bifidobacteria groups at baseline. Boxplots represent medians and interquartile range (IQR) with whisker end points equal to the maximum and minimum values below or above the median at 1.5 times the IQR.

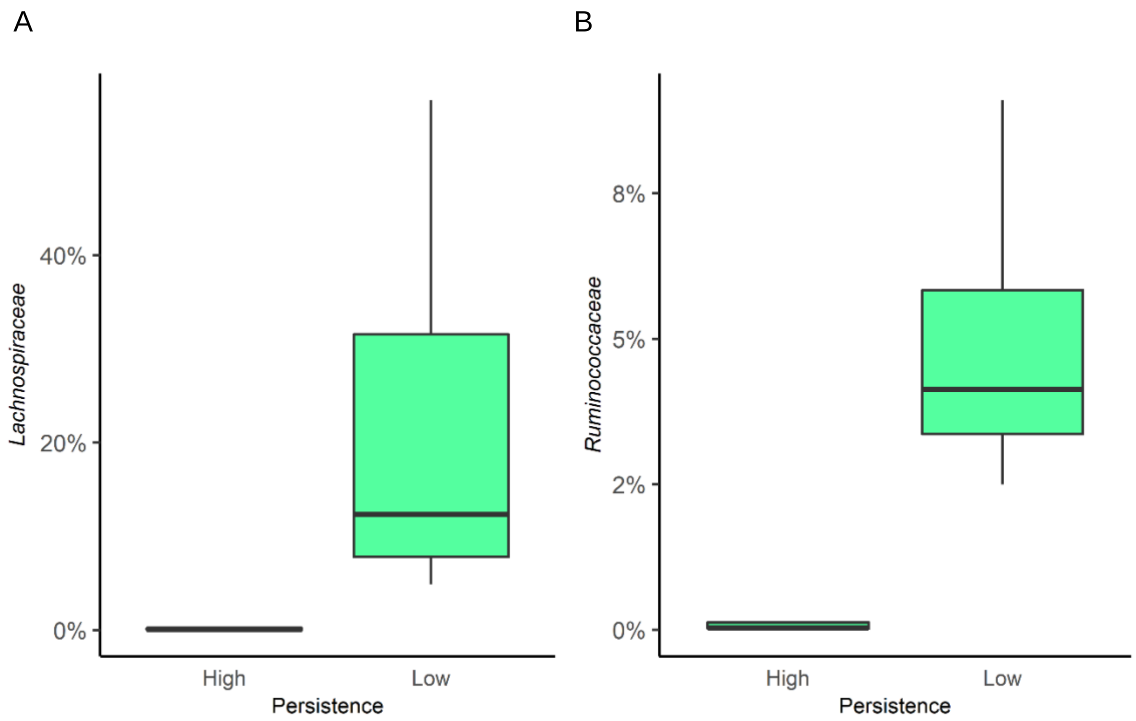

Supplementary Figure 2. Relative abundance of *Lachnospiraceae* and *Ruminococcaceae* at the final time point for 2'-FL treated mice grouped as highly enriched (HE) (n = 4) and moderately enriched (ME) (n = 7) *Bifidobacteriaceae* based on median relative abundance (50.5%). Boxplots represent medians and interquartile range (IQR) with whisker end points equal to the maximum and minimum values below or above the median at 1.5 times the IQR.
